# Surface Modification Using MAPLE Technique for Improving the Mechanical Performance of Adhesive Joints

**DOI:** 10.3390/nano13060964

**Published:** 2023-03-07

**Authors:** Valentina Dinca, Gabriela Toader, Raluca Gavrila, Oana Brincoveanu, Adrian Dinescu, Edina Rusen, Aurel Diacon, Alexandra Mocanu

**Affiliations:** 1 National Institute for Laser, Plasma and Radiation Physics, 409 Atomiștilor Street, 077125 Măgurele, Ilfov, Romania; 2 Military Technical Academy “Ferdinand I”, 39-49 Blvd. George Coșbuc, Sector 5, 501410 Bucharest, Romania; 3 National Institute for Research and Development in Microtechnologies IMT, 126A Erou Inacu Nicolae Street, 077190 Bucharest, Romania; 4 Faculty of Chemical Engineering and Biotechnologies, University Politehnica of Bucharest, Polizu Campus, 1-7 Gh. Polizu Street, Sector 1, 011061 Bucharest, Romania

**Keywords:** matrix-assisted pulsed laser evaporation (MAPLE), adhesive bonding, surface modification, thin film, polyvinyl alcohol, triethanolamine, aluminum plates

## Abstract

The adhesive bonds that ensure the appropriate mechanical properties for metal joining imply the surface chemical and wetting modification characteristics of the substrates. In this work, matrix-assisted pulsed laser evaporation (MAPLE) was used for the surface modification of Al via the deposition of two chemical compounds, polyvinyl alcohol (PVA) and triethanolamine (TEA), from frozen aqueous solutions. The deposition of the TEA and PVA layers was evidenced by FT-IR, SEM, and AFM analysis. The contact angle measurements evidenced the change in the hydrophilicity of the surface and surface free energies. The performance of the commercial silyl-based polymer adhesive Bison Max Repair Extreme Adhesive^®^ was evaluated by tensile strength measurements. This method led to a change in tensile strength of 54.22% in the case of Al-TEA and 36.34% for Al-PVA compared with the control. This study gives preliminary insights into using MAPLE, for the first time in adhesive applications, as a pretreatment method for Al plates for adhesive bonding reinforcement.

## 1. Introduction

Improving the mechanical properties of adhesive joints can require different techniques related to the preparation of the surface treatment of the substrate as well as the possibility of modifying the adhesive formulation to reinforce the interface bonding. 

In the automotive and aerospace industries, the need for lighter materials with mechanical performances similar to carbon steel has increased in the last decade. According to Cavezza et al. [1], the demand for aluminum in automobiles has doubled in the last 10 years when considering that aluminum-based components are 50% lighter. In the automotive business, more than 10 kg of adhesives may occasionally be needed to secure aluminum bonding, while the aerospace industry aims to cut aircraft weight by replacing riveting with adhesive bonding in the case of joining metal-to-metal parts or dissimilar materials [1,2,3]. The advantages of aluminum or aluminum-alloy in both industries are related to the high strength-to-weight ratio and increased resistance to corrosion. However, in terms of aluminum-to-aluminum bonding, the first steps for good mechanical performances in terms of fatigue or life-cycle assessments are related to the surface cleaning or pretreatment of the metal substrates [1,4,5], followed by the selection of commercial adhesives based on their chemistry [1,6,7] or the design of functionally graded adhesives [8].

In order to ensure a robust and durable joint, surface bombardment (activated plasma), mechanical, chemical, electrochemical, or laser action are some of the techniques that can be used to treat aluminum (Al) substrates in order to remove contaminants or dust from the surface or protect the substrate from corrosion [5,9,10]. Among all these methods, the chemical modification of the surface or adhesive formulation enhances the adhesive, mechanical performance through chemical bond formations between the functional groups of various organic/inorganic compounds (i.e., hydroxides, amines, silanes, etc.) that are compatible with a specific adhesive (i.e., acrylic, epoxide, etc.) [5,8,11,12,13].

Thin film deposition techniques have been applied in numerous fields, like electronics, coatings, packaging, or photovoltaic cells, using different methods such as sol-gel, hydrothermal synthesis, electrochemical methods, spray coating, spin coating, physical/chemical vapor deposition, laser-based techniques, or evaporation [14,15,16,17,18,19,20]. Most of these methods are predominantly related to the deposition of inorganic compounds as thin layers on different surfaces, being less applicable for generating organic substrates. Among the laser deposition techniques, the MAPLE deposition procedure was developed to reduce the drawbacks of physical vapor deposition (PVD) by avoiding the breakage of fragile materials during deposition and by ensuring a uniform, homogeneous film formation for solvent-based coatings [21]. Currently, MAPLE is one of the most versatile thin film deposition procedures for different organic, inorganic, polymer, or biological materials, providing great potential for the modification of various substrates, like polymers and metal plates, through the action of a laser beam for a large range of applications from optical and electro-optical to organic coatings for antimicrobial or biocompatible materials [22,23,24,25,26].

While methods such as spray coating, dip coating, or spin coating can provide fast and reproducible polymer/organic compound coatings, in terms of adhesion strength between the polymeric layer and the substrate, however, MAPLE was shown to provide superior adhesion on any type of substrate, with the coating following the profile of the substrate. In the case of spin coating, any defect on the surface would lead to more polymeric defects in the coating. Dip coating and spray coating might lack good enough adhesion to resist adhesion tests. Furthermore, the controlled thickness of the deposited layer cannot be guaranteed by spin coating or dip coating. By using MAPLE, layers (from a few tens of nm up to 1 micron) can be uniformly obtained by varying laser parameters (wavelength, fluence) and target parameters (i.e., solute, concentration). Nevertheless, the main advantage of MAPLE in the present discussed work is the ability to provide a layer with good adherence on the metallic surface for adhesive bonding.

In terms of adhesion or adhesive compounds, MAPLE has not been used so far for the pretreatment of metals for adhesive joints in the automotive, aerospace, or civil engineering domains, being exploited more for the manufacturing of organic or polymeric thin films in the field of electronic devices or biomaterials [27,28]. As a result, this study brings a novel approach in terms of the MAPLE applications in which this technique is used as a surface pretreatment of aluminum 6061-T6 alloy sheets (Al) for adhesive bonding, which is a first to our knowledge. Aluminum 6061-T6 sheets are used in certain structural applications that involve the manufacturing of boats, ships, furniture, etc., and are one of the most versatile aluminum alloys in industrial and commercial applications.

Due to the present greater cost of this positioning technology, one of our key goals is to provide an alternative for surface pretreatments of the Al substrate using the MAPLE process in order to increase the tensile strength of the bonded plates for special applications. However, for the moment, the scaling-up of this process will be a great challenge for applications in the automotive, naval, aerospace, or civil engineering industries. Thus, the novelty of this study consists of changing the wetting characteristics of Al substrates, which is one of the determining factors of the mechanical properties of the adhesive bonds, by employing MAPLE technique as a surface pretreatment method.

In order to demonstrate our hypothesis, uniform layers of PVA and TEA from aqueous solutions were deposited on an Al surface. These two different chemical compounds were chosen based on their differences in molecular weight and hydroxyl group content, considering that better compatibility between the Al plates and the commercial adhesive Bison Max Repair Extreme Adhesive^®^, Bison International B.V., The Netherlands can be obtained.

After the surface modification of Al, the plates were lap-jointed using Bison Max Repair Extreme Adhesive^®^, which is a silyl-based adhesive for universal bonding surfaces (metal, wood, etc.).

In conclusion, this study gives a preliminary insight into using MAPLE for the first time in adhesive applications as a pretreatment method regarding Al plates for adhesive bonding, reinforcing the fact that surface treatments are considered necessary for the enhancement of adhesive, mechanical properties when commercial adhesives are used and when there is no possibility to manufacture a customized product.

## 2. Materials and Methods

### 2.1. Materials

Aluminum 6061-T6 alloy sheets (Al) with 2 mm thickness were used for the joining specimens. Ethanol (Sigma-Aldrich, Bucharest, Romania) was used as such to clean Al before deposition. Polyvinyl alcohol (PVA) (molecular weight Mn: 124,000 g/mol) and triethanolamine (TEA) were purchased from (Sigma-Aldrich, Bucharest, Romania) and used without further purification. The specimens were bonded by silyl-based polymer commercial adhesive Bison Max Repair Extreme Adhesive^®^ for all types of materials (Bison International B.V., Goes, The Netherlands.)

### 2.2. Methods

#### 2.2.1. Preparation of PVA and TEA Solutions

Two aqueous solutions of 5% by wt. concentration of PVA and TEA were prepared. After complete solubilization of the organic compounds, the target formation involved the use of liquid nitrogen for the freezing process of the solutions in the copper holder. Afterward, the holder was mounted inside the deposition chamber while the targets were kept frozen during the whole deposition process by using liquid nitrogen.

#### 2.2.2. Deposition of PVA and TEA by MAPLE Technique on Al Plates

**Scheme 1:**
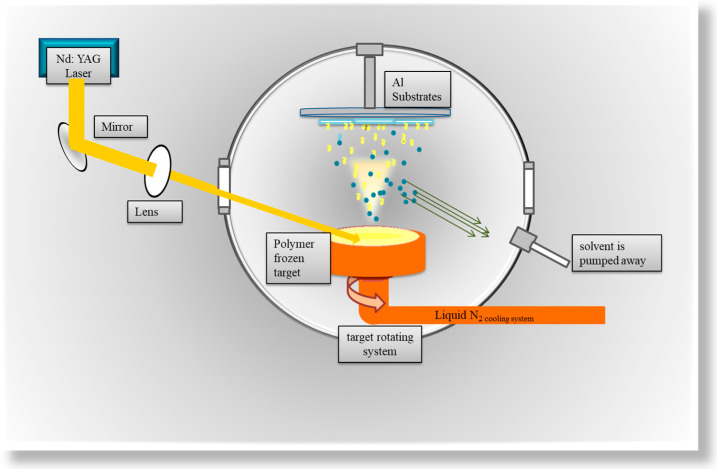
MAPLE set-up for obtaining the modified Al plates with PVA and TEA, respectively.

The cleaned Al plates (5 × 1 cm^2^; deposition area 1 × 1 cm^2^) were placed inside the chamber mounted on a holder placed at 3.5 cm above and parallel to the target as described in Scheme 1. Standard silicon plates were also used as deposition substrates for both organic compounds. To irradiate the frozen aqueous solutions of PVA and TEA (the targets), a Surelite II pulsed Nd:YAG laser system (Continuum Company, United States) working at a wavelength of 266 nm, with a 6 ns pulse duration and 10 Hz repetition rate was used. The laser fluence was 350 mJ/cm^2^, and the number of pulses was 72,000. The choice of fluence and the number of pulses was based on previous studies related to polymers and other sensitive compounds in terms of maintaining functional groups, as well as obtaining continuous and uniform coatings [29,30,31,32].

To avoid overheating of the target during irradiation, the target holder was rotated continuously (20 rpm). To evacuate the solvent, the deposition chamber was kept under a vacuum at a pressure of 1 × 10^−3^ Pa.

#### 2.2.3. Sample Preparation for Tensile Tests

After the deposition process, a thin layer of Bison Max Repair commercial adhesive was brushed on the PVA and TEA layers, respectively, on two distinct modified Al plates. The bonded specimens were kept at room temperature for 24 h for the reaction to be completed.

## 3. Characterization

### 3.1. Fourier-Transform Infrared Spectroscopy (FT-IR) of the Modified Al Plates

The infrared spectra of absorption for the two types of samples were obtained by FT-IR analysis, performed on a Spectrum Two FT-IR Spectrometer (Perkin Elmer, United States) equipped with a universal ATR—MIRacle™ Single Reflection ATR—PIKE Technologies at a 4 cm^−1^ resolution from 500 to 4000 cm^−1^ and a buildup of 32 scans.

### 3.2. Contact Angle Measurements

To determine the hydrophilic nature of the PVA and TEA layers and the surface free energy, the contact angles were measured with an EW-59780-20 Contact Angle Meter 110 VAC, 50/60 Hz. The contact angle measurements were determined by drop shape method using 3 μL water, respectively methylene iodide droplets. The contact angle was measured using the acquired images of a solvent drop at the points of intersection between the drop contour and the projection of the surface. The images were recorded every 5 s for 2 min. The results were recorded as the average of five measurements performed on each specimen.

### 3.3. Scanning Electron Microscopy Analysis for Blank and Modified Al Plates

The aspect of the blank and modified samples was recorded at 10 kV through a field emission gun scanning electron microscope (FEGSEM) Nova NanoSEM 630 (FEI) (Hillsboro, OR, USA).

### 3.4. Atomic Force Microscopy Analysis of the Blank and Modified Silicon Plates

To investigate the surface topography of the deposited film Atomic Force Microscopy (AFM) characterizations were performed in Intermittent-Contact mode with a Ntegra Scanning Probe Microscope (NT_MDT Spectrum Instruments, United States). Scans were conducted by employing medium stiffness probes (HA_NC by NT_MDT Spectrum Instruments, 3.4 N/m nominal spring constant). The root mean square roughness (Sq) was determined for the PVA and TEA layers deposited on the standard silicon plates taking into consideration 6 images with an area of 5 × 5 µm. Statistical parameters Skewness (Ssk) and Kurtosis (Sku) of the surface profile were subsequently computed from the acquired data based on Equations (1)–(3).

Root mean square roughness (Sq):

(1) $$\mathrm{Sq}={\left(\overset{N}{\underset{i=1}{\sum }}\left[\frac{{\left(h_{i}-\;\bar{h}\right)}^{2}}{N}\right]\right)}^{1/2}$$

Skewness of sample (Ssk):

(2) $$Ssk=\frac{1}{{\mathrm{NSq}}^{3}}\overset{N}{\underset{i=1}{\sum }}{\left(h_{i}-\;\bar{h}\right)}^{3}$$

Kurtosis (Sku):

(3) $$Sku=\frac{1}{NS_{q}^{4}}\overset{N}{\underset{i=1}{\sum }}{\left(h_{i}-\bar{h}\right)}^{4}$$

Ssk (skewness) expresses how symmetrical the surface profile is towards the mean plane. Ssk > 0 denotes the predominance of peaks, Ssk < 0 is indicative of the prevalence of valleys, whereas a normal (Gaussian) distribution results in Ssk = 0 [33]. Sku (Kurtosis) reflects the degree of sharpness of the surface peaks and valleys (how spiky its features are). Sku > 3 for “spiky” surfaces, Sku < 3 for “bumpy” surfaces, whereas Sku = 3 indicates a normal surface height distribution [33,34].

### 3.5. Mechanical Tests

To evaluate the maximal stress value at break tensile mechanical tests were achieved on a Titan 2 Universal Strength-Testing Machine equipped with a 600 N force cell. For this test, the blank and modified Al plates were bonded with Bison Max Repair adhesive on a surface of 1 × 1 cm^2^. The tensile tests were performed at a speed of 1 mm/min extension rate, starting with 50 mm jaw separation (plain jaw faces). For each type of sample, five specimens were analyzed, and the mean values were reported. To enable comparison between the samples, the mean values for each sample were plotted on the same graph.

## 4. Results

**Scheme 2:**
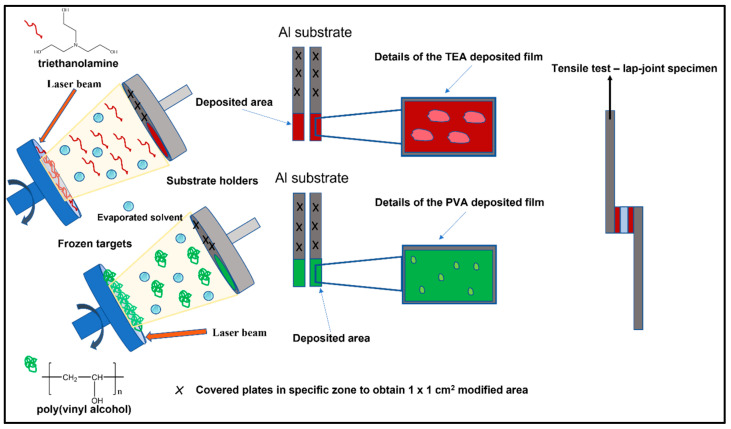
Applied MAPLE technique for surface pretreatment of Al plates for lap-joint tensile mechanical test.

The PVA and TEA layers were deposited on the Al plates using the MAPLE technique, according to the description method in Section 2.2.1 and Section 2.2.2, respectively. As is presented in Scheme 2, the Al plates were partially covered with tape (area marked with black X) to delimit the exact area of 1 × 1 cm^2^ necessary for the bonding step. The deposited area of PVA and TEA layers was further analyzed by FT-IR, contact angle measurement, SEM, and AFM to prove the advantage of Al surface pretreatment by MAPLE in the field of adhesives.

### 4.1. FT-IR Analysis of the PVA and TEA Deposited onto the Al Plates

FT-IR analysis was performed on standard silicon plates after the deposition of PVA and TEA, respectively, by the MAPLE technique, as described in Section 2.2.2. The FT-IR spectrum of the PVA layer sample showed a broad band at 3202 cm^−1^, attributed to the stretching vibration of a hydroxyl group with strong hydrogen bonding, while two small bands at 2380 and 2307 cm^−1^ were assigned to CH_2_ and CH stretching vibration, respectively. The stretching vibrational band of C=O was registered at 1709 cm^−1^ and was attributed to the carbonyl functional groups from the residual acetate groups of PVA. The bands observed at 1092 cm^−1^ were assigned to C-O stretching vibration [35].

For the TEA layer, a broad band at 3347 cm^−1^ was attributed to the hydroxyl group, while the peaks at 2812 cm^−1^ and 1341 cm^−1^ were due to the stretching and bending of C-H bonds. The peaks at 1057 cm^−1^ and 1010 cm^−1^ are attributed to C-O bond stretching, and the signal at 1655 cm^−1^ is due to the bending of the N-H group present in TEA [36]. The spectra of both layers are displayed in Figure S1 from the Supplementary file.

### 4.2. Contact Angle Measurements

In order to demonstrate the change in hydrophilicity and the surface free energy, contact angle measurements were performed via the sessile drop method for the blank and both of the specimens that were deposited onto the Al substrate using water and methylene iodide.

Surface free energy (SFE) or surface tension is composed of two components, the dispersive and the polar component, respectively, and are calculated according to Fowkes [37]; these and presented in Equation (5).

(4) $$\gamma =\gamma^{D}+\gamma^{P}$$

where γ is the SFE, $\gamma^{D}$ is the dispersive component, and $\gamma^{P}$ is the polar component, all expressed in mJ/m^2^.

In solid-liquid interactions, these two components can be determined based on contact angle measurements and by the system of Equations (5) and (6) (expressed below) for both liquids used to evaluate the contact angle measurement [38].

(5) $$\gamma_{L1}\cdot \left(1+\cos\left(\theta_{1}\right)\right)=2\cdot \left(\sqrt{\gamma_{S}^{D}\cdot \gamma_{L1}^{D}}+\sqrt{\gamma_{S}^{P}\cdot \gamma_{L1}^{P}}\right)$$

(6) $$\gamma_{L2}\cdot \left(1+\cos\left(\theta_{2}\right)\right)=2\cdot \left(\sqrt{\gamma_{S}^{D}\cdot \gamma_{L2}^{D}}+\sqrt{\gamma_{S}^{P}\cdot \gamma_{L2}^{P}}\right)$$

The values of SFE, as well as the dispersive and polar components for both water (L1) and methylene iodide (L2), were selected from the *Table of Common Numbers in Physics* [39] and shown in Table 1.

In order to give a more detailed characterization of the interfacial compatibility between the Al plates and the PVA and TEA layers, respectively, deposited onto the surface of the blank samples, adhesion work (W_a_) was calculated from the experimental values of the contact angle by considering the Young-Dupré equation [40]:

(7) $$W_{a}=\gamma_{L}\cdot (1+\cos\left(\theta \right))$$

Thus, Table 2 summarizes the values of the contact angle, surface free energy respective to the adhesion work obtained for all samples, while the droplet spreading effect of the blank and modified samples is presented in the Supplementary file—Figure S2.

### 4.3. SEM Analysis of Al and Modified Al Plates

**Figure 1:**
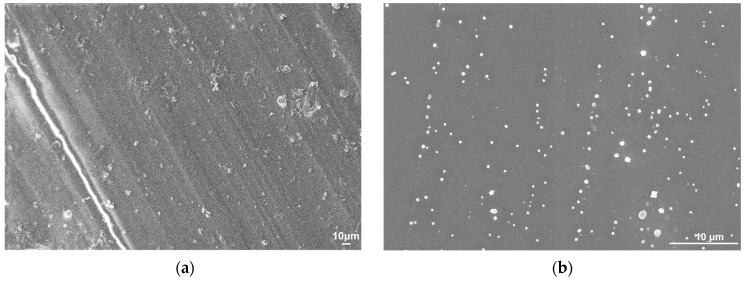

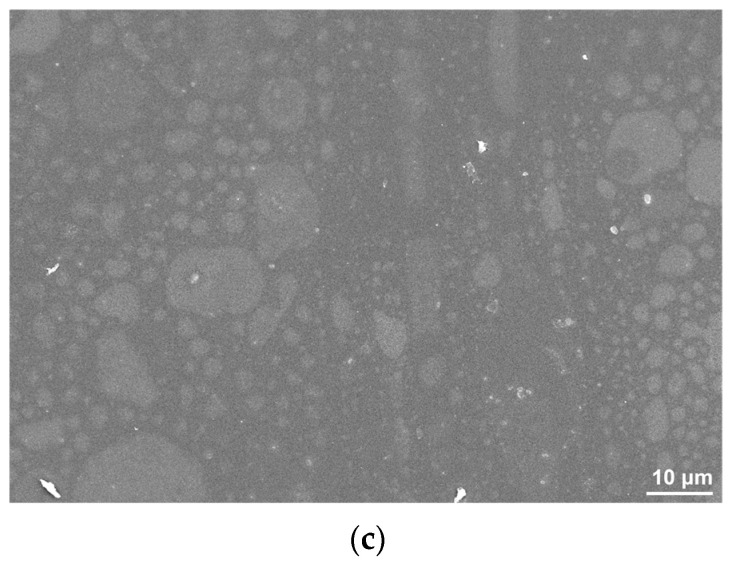
SEM micrographs for blank Al substrates (**a**) and modified Al plates with PVA (**b**) and TEA (**c**), respectively, via the MAPLE technique; scale bar: 10 μm.

The SEM micrographs presented in Figure 1 demonstrated the deposition of continuous layers (by MAPLE) with small aggregates in both cases for PVA and the island-like structures for TEA.

From Figure 1b, it can be seen that relatively small and uniform “dots” of PVA formed on the surface of the Al substrates, while nonuniform, island-like structures of TEA, ranging from 2 to over 20 μm, formed (Figure 1c). These results could explain the lack of several characteristic peaks that were not detectable by FT-IR analysis from the very thin layer (probably formed on the surface) in different areas of the samples.

### 4.4. AFM Analysis of the Deposited Organic Compounds

For imaging and quantitatively assessing the morphology of the deposited layers by AFM, the substrate needs to be smoother than the layers of concern. This prerequisite does not hold true for the Al substrates used in the experiments, which have a much higher roughness (>100 nm rms) than the layers deposited by MAPLE (see Figure S3 from Supplementary file). Therefore, for AFM imaging purposes, we have prepared a concurrent set of specimens on polished Si substrates under the same conditions as the Al substrates. The polished Si, with a roughness on the order of 0.2 nm rms is an ideal substrate for AFM measurements of common thin films.

AFM analysis confirmed the results previously obtained by SEM, namely the formation of small aggregates in the case of PVA and of larger ones for TEA.

In order to get a much clearer picture of the surface profile, the statistical parameters of the surface texture, namely root mean square roughness (Sq), skewness (Ssk), and kurtosis (Sku), were further computed from the AFM data using the image processing software. The values of these parameters were obtained based on Equations (1)–(3) [33,34].

**Figure 2:**
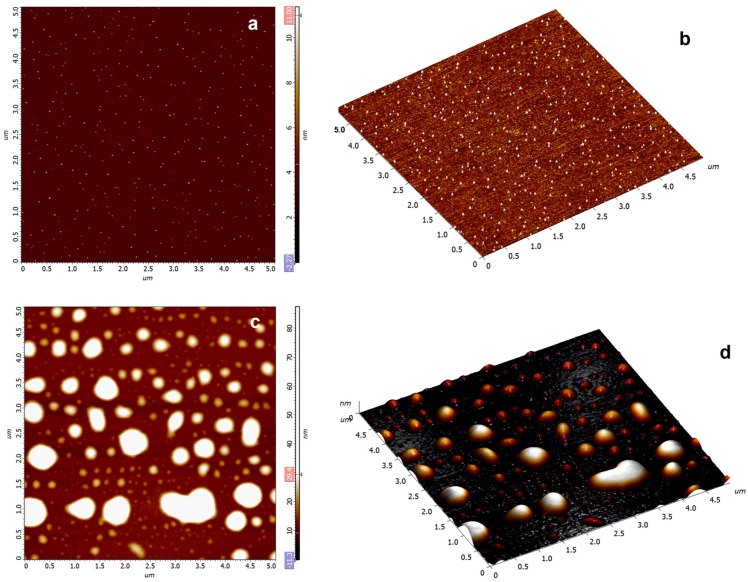
AFM images of PVA (**a**,**b**) and TEA (**c**,**d**) after deposition on Si plates by MAPLE, acquired as 2D and 3D renders, respectively. Scan size: 5 μm.

Thus, Figure 2 shows illustrative AFM images (scan size: 5 µm) of the PVA and TEA compounds deposited on the Si substrates, while Table 3 gives the mean values of Sq, Ssk, and Sku.

### 4.5. Mechanical Tests

**Figure 3:**
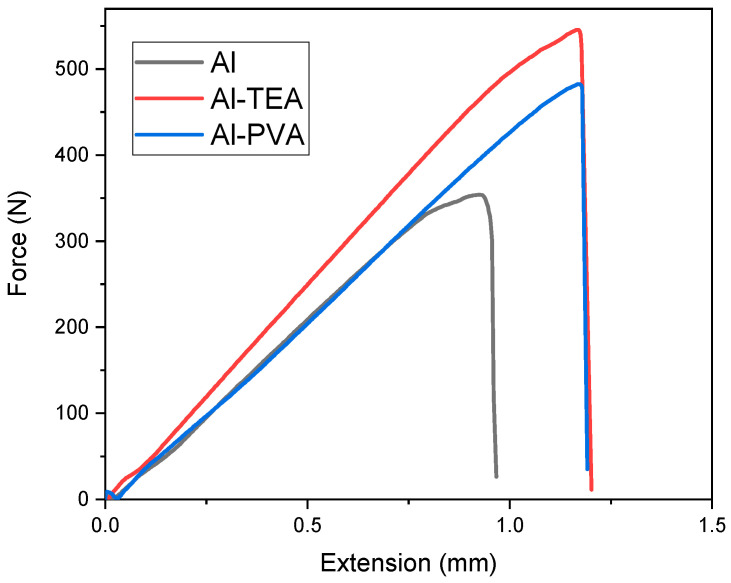
Tensile test measurements for the Al (blank), Al-PVA, and Al-TEA samples bonded by Bison Max Repair commercial adhesive.

Figure 3 illustrates the mean values obtained for the glued metallic coupons subjected to the tensile test. The samples utilized for the tensile test (blank Al, Al-PVA, and Al-TEA) were bonded by employing the silyl-based polymer commercial universal adhesive Bison Max Repair Extreme Adhesive^®^, as described in Methods section.

In order to compare the results of the tensile tests, two parameters were taken into consideration: the force constant (k) and the area under each curve obtained by the integration of each plot (A). The values of k for each specimen were calculated on the linear region of the plots, according to Equation (8).

(8) $$k=\frac{F\;}{x}$$

The force, *F* (expressed in N), is the maximum force reached by the samples before detaching, and x is the corresponding elongation (mm).

Parameter k is a measure of the stiffness of the material. At the same time, the values obtained for A are related to the energy stored by the tested material at the applied force necessary to deform an elastic object (also referred to as elastic potential energy or deformation energy). Thus, the area under the stress-strain curves in Figure 3 describes the energy stored by the samples bonded with the polymeric adhesive until the coupons are detached (until the force is removed). Fmax was also assessed by tensile tests, indicating the maximum force measured at the moment of separation of the two bonded metal plates. Table 4 gives the mean values for each sample and parameter.

## 5. Discussion

Our first aim of this study was to analyze the comparative adhesion performances of the two compounds with different molecular weights and different hydroxyl group contents. In terms of the chemical composition of the selected commercial adhesive, silyl-based polymer adhesives possess hydroxylic groups capable of forming hydrogen bonds for better compatibilization. Thus, the first approach of this study implied the use of a polymer compound and a small molecular organic chemical to analyze the differences in terms of adhesion improvement between the substrate, the deposited layers, and the commercial adhesive. Thus, we took into consideration that, in the case of TEA, the three hydroxyl groups represent 34.18% wt. of the molecular weight, while in the case of PVA, the hydroxyl group content calculated from the structural unit increases to 38.16% wt.

The first step in this study involved the modification of the Al plates and the investigation by FT-IR analysis in order to detect the characteristic vibrational signals of both organic compounds deposited on the standard silicon substrate. The results from the previous section confirmed the particular peaks for each compound deposited onto the Al plates. However, it is worth mentioning that some of the characteristic signals of the two compounds mentioned in the literature data were not detected by FT-IR analysis, probably due to the very thin layer deposited on the surface.

The surface treatments for the metallic substrates are sometimes crucial in adhesive bonding and in the improvement of mechanical performances of the joints. Thus, by using contact angle measurements, the determination of surface free energies (SFE) and adhesion work (Wa) demonstrated the surface modification of the Al plates (data shown in Table 2).

Al is a hydrophilic material, demonstrated by a contact angle value of 88.4° [41]. In order to achieve strong adhesive bonds, in our case, the purpose was to increase the hydrophilic character of the Al plates by creating uniform hydrophilic layers that were more compatible with the silyl-based polymer Bison Max Repair Extreme Adhesive and with the substrate. This approach is based on scientific data intensively presented by other researchers that applied different surface pretreatments to create a stronger bond between the hydrophilic groups of the coated layer and polar groups from the adhesive formulation [9,42].

By using the MAPLE deposition technique, we expected the formation of a continuous layer in the case of TEA, ensured by the laser’s number of pulses. However, the AFM results revealed that TEA was noncontinuous and island-like structures were formed (Figure 2d). Nevertheless, the wettability study of TEA for discontinuous structures is presented in the literature data [43].

The modified Al substrate showed good wettability in both cases, confirming, as expected, increased hydrophilicity for both Al-PVA and Al-TEA compared with the blank specimen. Thus, the contact angle of the Al plates decreased from 88.4° to 78.45° in the case of Al-PVA, and 50.07° for Al-TEA in the case of the contact angle measurements performed for water (Table 2). This result was attributed to the hydroxyl groups present in the chemical structure of both compounds.

In order to correlate the adhesion phenomena between the substrate and the deposited layer, the SFEs of all samples were determined based on the contact angle values registered for water and methylene iodide (Table 2). SFE is a parameter that gives quantitative information about the adhesion phenomena between the blank Al sample and the deposited layers of PVA and TEA. Thus, the values obtained in the case of TEA (53.65 mJ/m^2^) and PVA (28.74 mJ/m^2^), respectively, were higher compared to the blank (28.27 mJ/m^2^). Practically, in the case of TEA, the value of SFE almost doubled compared with the unmodified substrate. This increase in the surface energy correlated with the decrease in the contact angle and could be an indication of better compatibility between the two systems, which could bring an improvement in adhesive bonding. However, to foresee such an improvement in adhesion, Wa is another trustworthy indicator that shows the system’s compatibility [38]. Thus, the higher the values of Wa, the better stability and compatibility between the Al substrate and the deposited layers.

As expected from the values obtained for the SFE, in the case of Wa, we registered the same trend in which the value of Wa for the Al-TEA sample registered 119.52 mJ/m^2^, which is much higher compared to the blank sample (74.83 mJ/m^2^). Additionally, for Al-PVA, the value of Wa increased to 87.3 mJ/m^2^, proving good interactions between the Al plates for both compounds. Thus, by registering these values in the case of Wa, we expect a stronger bonding as a consequence of the surface pretreatment.

Our next step in characterizing the deposited layers consisted of AFM statistical analysis to determine the Sq parameter, as well as the Ssq and Sku values (Figure 2) performed on the Si plates, as explained in Section 4.4. The AFM statistical parameters computed from the image processing software and presented in Table 3 revealed interesting information for both the deposited layers. As was expected from the SEM analysis, the PVA layer has a much lower Sq (0.9 nm) value compared to TEA (13.1 nm). Both sample profiles feature a predominance of peaks (Ssk > 0), but the profile asymmetry is far more pronounced for PVA (considerably higher Ssk value) (Table 3). Furthermore, the PVA layer morphology exhibits a strong, spiky character (Sku value of 25.6) compared to the more flat-bottomed morphology of the TEA (Sku = 11.1).

This is also in perfect accordance with the findings of the SEM scans (Figure 1), namely the presence of small, scarce aggregates for the PVA sample and more uniform morphology and the island-like structures in the case of the TEA sample.

The last step in the characterization of this study implied the demonstration of the anticipated performance of the adhesive bond from the data registered based on SFE and Wa evaluation. When the results from Table 4 were compared, both of the samples modified by MAPLE saw an improvement in mechanical strength compared to the reference. Al-TEA recorded the highest values for A and Fmax, with an increase of 87.34% for A and 54.22% for Fmax, respectively, while Al-PVA had an increase of 60.2% for A and 36.34% for Fmax when compared to the blank sample. These results are in good agreement with the SFE and Wa values since these values increased for both the deposited layers when compared to unmodified Al. Furthermore, the higher values of A and Fmax obtained in the case of the Al-TEA specimens can be correlated with the Sku value of TEA layer, which was considerably lower when compared to the PVA analogous samples.

As mentioned before, the k value is an indication of the stiffness of the materials. Thus, the values obtained for the k parameter allowed us to provide a more precise interpretation of the mechanical performance of the bonded structure. Thus, by increasing the value of k by 7.2% for the Al-modified with PVA and 22.32% for Al-TEA, more rigid structures were formed. Besides, the k value confirms that the reference and Al-PVA samples possess a lower stiffness compared with Al-TEA.

**Scheme 3:**
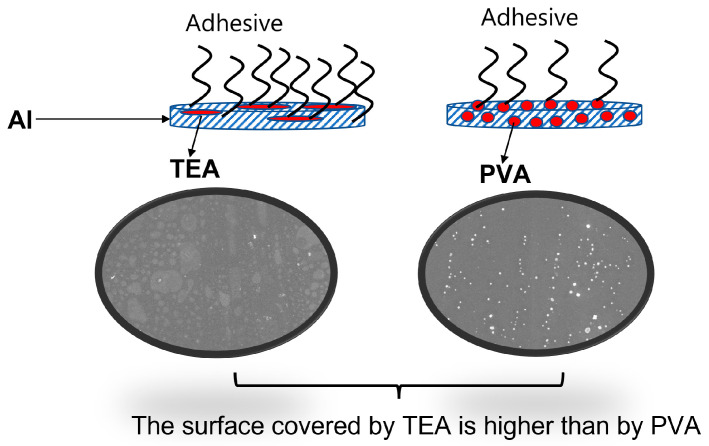
Possible orientation of the PVA or TEA structures between the Al substrate and the adhesive layer.

Taking into consideration the purpose of this study, the above-mentioned theoretical value in terms of hydroxyl group content in the case of PVA (38.16% wt.) is higher, while the hydrophilicity, SFE, and Wa of the TEA layer deposited on the Al plates proved to be greater, which led to better compatibility between the substrate and the adhesive. Thus, most likely, the -OH groups are facing the adhesive, whereas the main chain of PVA, or the -N groups of TEA, are facing the aluminum plates (Scheme 3).

In the case of the metal preparation techniques for improving adhesive bonding, classical pretreatment techniques might sometimes have limitations due to the environmental or health risks associated with degreasing or chemical cleaning procedures or due to a lack of confidence in the reproducibility of mechanical treatments (i.e., abrasion, sand-blasting, etc.) [44]. Another issue in terms of the practical requirements for long-lasting adhesive bonding relates to the wettability of the surface that promotes the spreading of the adhesive for better compatibility between the substrate and the bonding layer. Thus, diverse surface pretreatments (of the substrates) can be used to ensure wettability modifications for metal bonding [44,45]. As an alternative to cleaning or modifying the surface morphology of metal parts before joining, laser irradiation is employed in the automotive and aerospace industries [44].

Almost two decades ago, Spadaro et al. [46] investigated the surface modifications of aluminum alloys after irradiation with an Nd-YAG laser using a 532 nm wavelength at different repetition rates and energy densities to remove surface contaminants on the one hand and produce morphology changes on the other to improve bonded joints. Laser irradiation at this time was intended to replace a series of subsequent steps applied in the cleaning of metal substrates before bonding, like degreasing, mechanical grinding, acid treatment, and anodizing processes regarding Al plates. Hence, the mechanical characteristics of the bonding were enhanced by introducing “defects” ascribed to the variations at the microstructure level produced by the greater energy densities of the laser.

Similar results were obtained by Rechner et al. [47], who proposed laser irradiation as a dry pretreatment method for the aluminum alloys used in car bodies as an alternative for the removal of all types of impurities and registered an improvement in tensile shear strength of up to 20%.

Related strategies were applied by Rotella et al. [48], in which steel was pretreated by pulsed laser irradiation at a wavelength of 1064 nm, leading to surface texture changes that modified the mechanical performances of the bonded plates used in the automotive industry. As a result, the peel strength was significantly increased when comparing the laser irradiation approach to degreasing and sandblasting, while the shear stress of the joint did not register any appreciable changes.

Recent research by Boutar et al. [45] showed that, following laser irradiation, lap shear strength increased by up to 22% and even 50% when compared to the reference metal substrates that had been chemically cleaned. Moreover, they demonstrated that the shear strength of the surface-modified substrates could be increased by reducing the contact angle, a finding that is also in line with our findings.

In our case, using the MAPLE technique, the pretreatment involved surface modification through the deposition of two compounds that, on the one hand, decreased the contact angle and, on the other, improved tensile tests, registering an increase in tensile strength of 36.34% for Al plates modified with PVA, respectively, of up to 54.22% for Al-modified with TEA. To conclude, we were able to acquire results using this new laser technology that was in strong agreement with research that has already been published. Thus, the traditional chemical, mechanical, or other laser-irradiation processes required for the surface modification of aluminum plates could be replaced by this laser method.

Once more, our hypothesis was proven to be correct by the contact angle measurements, SFE, Wa determination, SEM, and AFM analysis, which showed that the compound with the smaller molecular weight produced superior adhesion between the Al plates and Bison Max Repair Extreme Adhesive.

Thus, according to the results obtained from this study, we can affirm that the MAPLE technique significantly improved the mechanical properties by changing the characteristics of the surface of the Al plates, promoting better performances for the adhesive formulation.

## 6. Conclusions

Al plates were modified via the MAPLE technique with PVA and TEA aqueous solutions of 5% wt. The deposited layers were analyzed by FT-IR, contact angle measurements, SEM, and AFM. Some of the characteristic FT-IR signals of the two layers mentioned in the literature data were not entirely detected due to the very thin layer that was deposited on the surface of the aluminum plates. The contact angle measurements revealed that the layers led to a more hydrophilic surface on the modified Al plates, while SFE and Wa indicated higher compatibility between the two layers and the Al substrate. The SEM and AFM analysis demonstrated that the PVA and TEA layers deposited by MAPLE were homogeneously distributed, being dominated by small aggregates in the case of PVA and island-like structures in the case of TEA. However, the mechanical tests revealed an increase in Fmax for the commercial adhesive (Bison Max Repair Extreme Adhesive^®^) of 36.34% for Al-PVA and 54.22% for Al-TEA, respectively, and an increase in stiffness for both samples in comparison with the reference. Thus, through this study, we proved the importance of surface modification to maximize the mechanical performances of commercial adhesives for future applications in the automotive, naval, and aerospace industries. However, at this time, scaling up this method remains a challenge, although the premise, in terms of mechanical performances, is quite promising. We believe that the field of electronic devices could benefit from this strategy also.

## Figures and Tables

**Table 1:** The values of SFE and the dispersive and polar components of water and methylene iodide.

Liquid	γ_L_ (mJ/m^2^) *	$\gamma_{L}^{D}\;(\mathrm{mJ}/m^{2})$	$\gamma_{L}^{P}\;(\mathrm{mJ}/m^{2})$
Water	72.8	21.8	51.0
Methylene iodide	50.8	50.8	0

**Table 2:** Contact angle, surface free energy, and adhesion work values for the Al and modified Al plates.

Sample	Contact Angle Values for Water	Contact Angle Values for Methylene Iodide	Surface Free Energy (SFE) (mJ/m^2^)	Adhesion Work (W_a_) (mJ/m^2^)
Al	88.4 ± 1.2°	67.74 ± 0.28°	28.27	74.83
Al-PVA	78.45 ± 1.3°	80.94 ± 1.06°	28.74	87.3
Al-TEA	50.07 ± 4.13°	55.16 ± 1.97°	53.65	119.52

**Table 3:** Statistical Summary for the roughness parameters of PVA and TEA deposited by MAPLE on polished Si.

	Sq (nm)				Ssk				Sku			
Deposited Layer	Mean	Std. Dev.	Min	Max	Mean	Std. Dev.	Min	Max	Mean	Std. Dev.	Min	Max
PVA	0.9	0.2	0.6	1.1	3.2	1.8	1.6	6.6	25.6	22.4	12.8	70.9
TEA	13.1	2.0	9.5	15.2	2.3	0.3	1.8	2.7	11.1	1.9	8.5	14.1

**Table 4:** Values of k, A, Fmax and the extension for the blank and Al-modified plates.

Sample	k, N/mm	Area N·mm	Fmax N	Extension (x), mm
Al	384.62 ± 2.83	185.87 ± 2.81	353.85 ± 2.87	0.92 ± 0.022
Al-PVA	412.35 ± 2.46	297.77 ± 2.43	482.45 ± 2.54	1.17 ± 0.015
Al-TEA	470.45 ± 2.14	348.22 ± 2.15	545.72 ± 2.08	1.16 ± 0.012

## Data Availability

Not applicable.

## Supplementary Materials

The following supporting information can be downloaded at: https://www.mdpi.com/article/10.3390/nano13060964/s1, Figure S1: FT-IR spectra of PVA (dark line), respectively TEA (red line) layers deposited by MAPLE; Figure S2: Contact angle measurements for blank Al and modified Al plates with PVA, respectively TEA in the presence of water (a, b, c) and methylene iodide (d, e, f); Figure S3: AFM cross-section (a) and 3D image for Al plates; Video S1: Matrix Assisted Pulsed Laser Evaporation (MAPLE) deposition technique, https://www.youtube.com/watch?v=C6F-qD8iuaU&t=1s.
