# Supplementary material for: Surface Modification Using MAPLE Technique for Improving the Mechanical Performance of Adhesive Joints
Source: Nanomaterials (Basel). 2023 Mar 7;13(6):964. doi: 10.3390/nano13060964 (PMC10054649; doi:10.3390/nano13060964)
Supplement: Supplementary file 1 [file nanomaterials-13-00964-s001.zip › nanomaterials-2207066-supplementary.pdf]

Surface Modification Using MAPLE Technique for Improving Mechanical Performance of Adhesive Joints

Valentina Dinca[1], Gabriela Toader[2], Raluca Gavrila[3], Oana Brincoveanu[3], Adrian Dinescu[3], Edina Rusen[4], Aurel Diacon[2,4], Alexandra Mocanu[4,]*

[1]National Institute for Laser, Plasma and Radiation Physics, 409 Atomiştilor Street, , 077125, Magurele, Ilfov, Romania

[2]Military Technical Academy "Ferdinand I", 39-49 Blvd. George Coşbuc, Sector 5, 501410, Bucharest, Romania

[3] National Institute for Research and Development in Microtechnologies IMT, 126A Erou Inacu Nicolae Street, 077190 Bucharest, Romania

[4]University Politehnica of Bucharest, Faculty of Chemical Engineering and Biotechnologies, 1-7 Gh. Polizu Street, Polizu Campus, Sector 1, 011061, Bucharest, Romania

*Corresponding author: alexandra.mocanu@upb.ro

Supplementary file

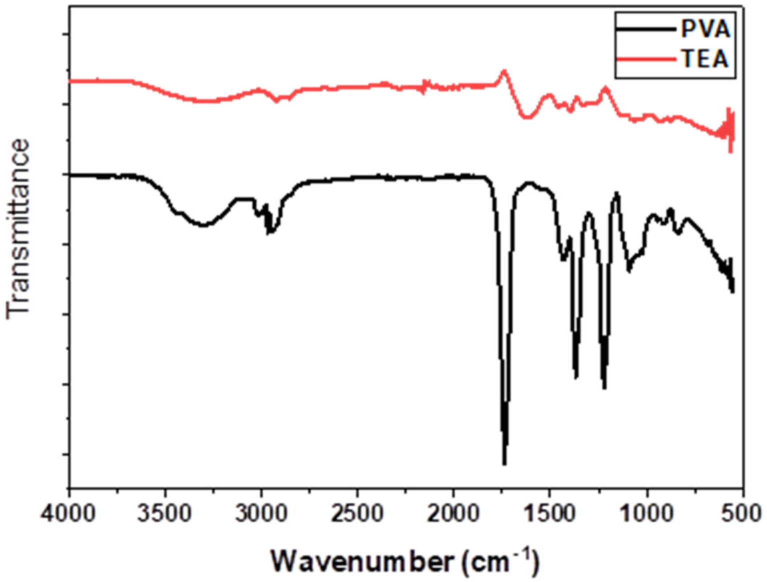

Figure S1. FT-IR spectra of PVA (dark line), respectively TEA (red line) layers deposited by MAPLE

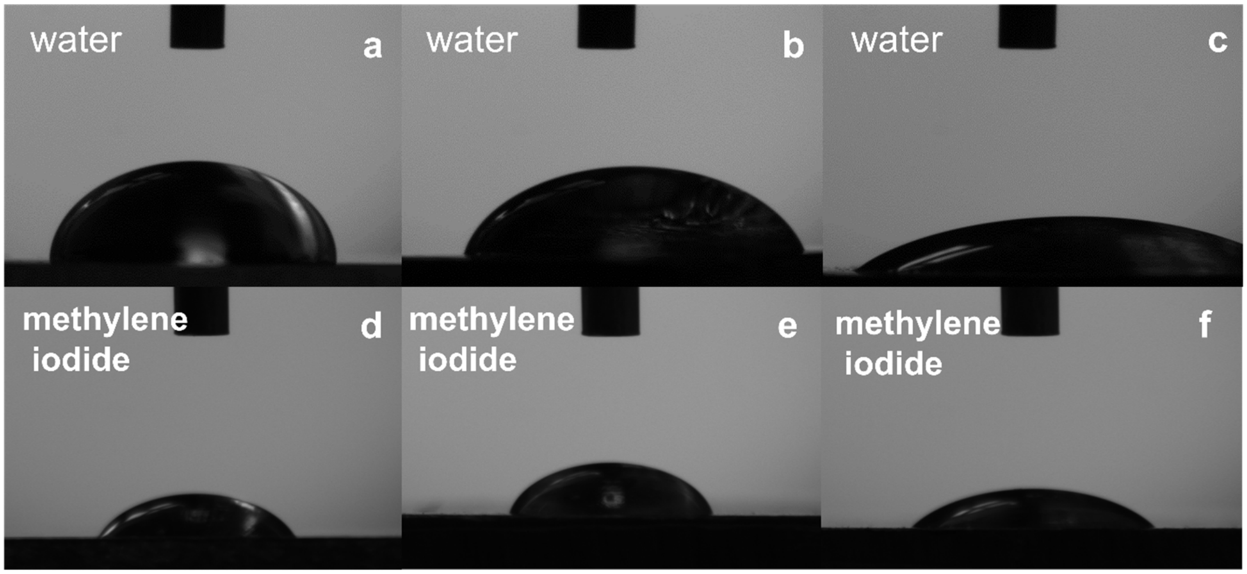

Figure S2. Contact angle measurements for blank Al and modified Al plates with PVA, respectively TEA in the presence of water (a, b, c) and methylene iodide (d, e, f)**.**

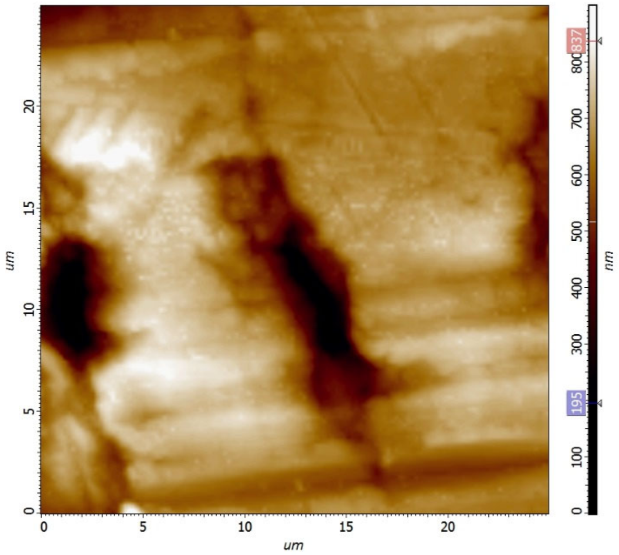

(a)

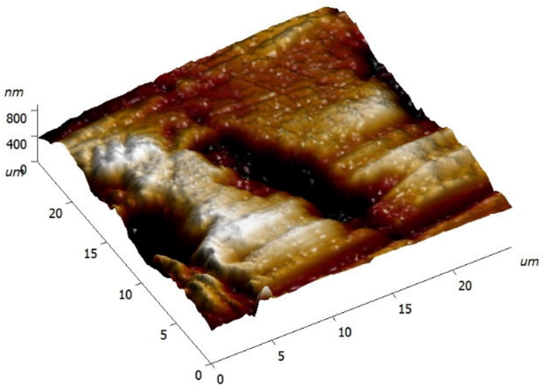

(b)

Figure S3. AFM cross-section (a) and 3D image for Al plates
